# Protective Effects of Salidroside on Mitochondrial Functions against Exertional Heat Stroke-Induced Organ Damage in the Rat

**DOI:** 10.1155/2015/504567

**Published:** 2015-11-18

**Authors:** Wei Zhang, Ming Peng, Yang Yang, Zhangwu Xiao, Bin Song, Zhaofen Lin

**Affiliations:** ^1^Department of Emergency, The Second Military Medical University Affiliated Fuzhou General Hospital, Fuzhou, Fujian 350025, China; ^2^Department of Emergency, Fuzhou General Hospital, Fuzhou, Fujian 350025, China; ^3^Medical Department, Fuzhou General Hospital, Fuzhou 350025, China; ^4^Emergency Department, Shanghai Changzheng Hospital, Shanghai 200003, China

## Abstract

Exertional heat stroke (EHS) results in a constellation of systemic inflammatory responses resulting in multiorgan failure and an extremely high mortality. The present study was designed to evaluate the protective effects of salidroside on EHS by improving mitochondrial functions in the rat model. Liver and heart mitochondria were observed by transmission electron microscopy and mitochondrial membrane potential (ΔΨm) was detected by a fluorescent probe. Intramitochondrial free Ca^2+^ concentration, mitochondrial respiratory control ratio (RCR), reactive oxygen species (ROS) levels, superoxide dismutase (SOD), and malondialdehyde (MDA) activity were detected by the corresponding kits. RT-PCR was performed to estimate peroxisome proliferator-activated receptor-*γ* coactivator-1*α* (PGC-1*α*) and manganese form of SOD (MnSOD) mRNA expression. The results demonstrated that salidroside was able to relieve EHS damage by reducing the swelling of mitochondria, ROS levels, and MDA activity, as well as increasing ΔΨm, RCR, free Ca^2+^ concentration, SOD, PGC-1*α*, and MnSOD mRNA levels. In conclusion, salidroside has protective effects on mitochondrial functions against exertional heat stroke-induced organ damage in the rat.

## 1. Introduction

There are two types of heat stroke: classic, nonexertional heat stroke (NEHS) and exertional heat stroke (EHS). Classic heat stroke occurs most commonly in very young or older individuals who have health risks and are in poor environmental conditions. Exertional heat stroke occurs more often in younger, healthy individuals who participate in strenuous physical activity. EHS is characterized by hyperthermia, central neurologic disorders, and variable levels of rhabdomyolysis during, or after, an intense or prolonged physical exercise [1]. EHS is a serious disorder that can be fatal especially if proper assessment and treatment are not initiated rapidly [2]. Apart from high temperature, hypoxia and oxidative damage may be responsible for heat stroke. Both high temperature and hypoxia could impair mitochondrial function, aggravate the body's hypoxia, and lead to further deterioration [3]. Heart and liver tissues are tissues with high and fluctuating energy demands, so they were chosen for analysis in this study.

Appropriate regulation of mitochondrial biogenesis and function is a critical component of adaptation to external conditions and prevention of pathogenesis. Mitochondrial dysfunction includes mitochondrial swelling, ΔΨm decrease, ROS and MDA increase, and antioxidant enzyme SOD reduction [4].

Mitochondria are key regulators of cell death following alterations in ΔΨm in response to various triggers. Loss of ΔΨm is a marker of mitochondrial dysfunction that ultimately leads to apoptosis. The respiratory control ratio (RCR) is a measure of the “tightness of coupling” between electron transport and oxidative phosphorylation. Deregulation of intraneuronal Ca^2+^ is known to generate high levels of reactive oxygen species of mitochondrial origin (mt-ROS), a crucial step in the excitotoxic cascade. An imbalance between production of ROS, oxidative damage to lipids (lipid peroxidation), and its elimination by the antioxidant defense system (such as SOD) in the body has been implicated for causes of aging and mitochondria related disease [4].

Timely protection of mitochondrial function helps to reduce EHS injuries to the body. Salidroside, a phenylpropanoid glycoside isolated from* Rhodiola rosea*, has been reported to have various pharmacological properties, including attenuating myocardial ischemia-reperfusion injury via PI3K/Akt signaling pathway [5], stabilizing the mitochondrial membrane potential, and inhibiting cell apoptosis by inhibiting hypoxia/hypoglycemia-induced mitochondrial membrane potential damage and reduced activity [6]. In addition, salidroside has been used as an antioxidative therapy for oxidative injury in cardiac diseases and protected against hydrogen peroxide-induced injury in HUVECs via the regulation of REDD1 and mTOR activation [7]. The purpose of this study was to determine whether salidroside could alleviate the symptoms of EHS by protecting mitochondrial functions.

## 2. Materials and Methods

### 2.1. Ethics Statement

All studies involving animals were carried out according to guidelines put forth by the institutional guidelines for the humane treatment of laboratory animals, as approved under protocol #IACUC-2012-009 by the Experimental Animal Ethics Committee of Academy of Military Medical Sciences.

### 2.2. Animals

Male Sprague-Dawley (SD) rats weighing 190–210 g (8–12-week-old) were obtained from the Animal Center of the Vital River. They were specific pathogen-free animals (SPFA) housed in clean, temperature- and humidity-controlled SPFA chamber with a room temperature of 22 ± 2°C, a relative humidity of 50 ± 5%, unlimited food and water, and a 12 h light/dark cycle.

### 2.3. Animal Model and Experimental Design

A total of 80 specific pathogen-free (SPF) SD rats were randomly divided into 8 groups (*n* = 10 per group): control group (Group A), exercise group (Group B), high temperature group (Group C), EHS group (Group D), salidroside low dose group (Group E), salidroside middle dose group (Group F), and salidroside high dose group (Group G). In Group A, rats were fed with normal standard method. In Group B, in the SPF animal room temperature (22°C), rats were running on a treadmill (treadmill speed: 25 rpm) and were given a shock (voltage 100 V) when they did not run; after the animals rested, they continued to cycle until exhaustion. In Group C, the thermostat inside temperature was adjusted to 45°C; rats do not exercise until exhaustion. In Group D, the thermostat inside temperature was adjusted to 45°C, with rats running according to the first set of Group B. Rats only received salidroside in the absence of heat or exercise (Group H, *n* = 10) to explore whether the effect of salidroside was specific to heat stroke or a nonspecific effect that causes Ca^2+^ increase and other responses regardless of whether one has heat stroke or not. The animals were sacrificed and the tissues were taken when they stopped running. Salidroside (with different doses, 4 mg/kg, 10 mg/kg, and 25 mg/kg, resp.) was administered into rats by gavage [8].

### 2.4. Mitochondria Isolation

Mitochondria of rat's heart and liver tissues were isolated using a mitochondria isolation kit according to the manufacturer's instructions (Pierce, Rockford, IL, USA) [9]. Briefly, samples were pelleted at 850 ×g for 2 min by centrifugation before mitochondria isolation reagents A, B, and C were added in the correct sequence. The mixture was centrifuged at 700 ×g for 10 min at 4°C. The supernatant was transferred to a new, 2.0 mL tube and centrifuged at 12,000 g for 15 min at 4°C. Mitochondria isolation reagent C was added to the pellet and centrifuged at 12,000 ×g for 5 min before the pellet was resuspended in 80 mmol/L sucrose with 0.1% bovine serum albumin (BSA). Mitochondrial fractions were kept at 4°C and studied within 3 h of isolation. A protein content assay (Bio-Rad, Hercules, CA, USA) was performed on the isolated mitochondria fractions.

### 2.5. Transmission Electron Microscopic Observation

Electron photomicrographs for hepatocytes and cardiomyocytes were prepared as described [9]. In brief, cells were washed with PBS and fixed in 4% glutaraldehyde/1% paraformaldehyde for 2 h at room temperature and postfixed in 1% OsO_4_ for 1 h at 4°C. After dehydration in graded ethanols (70%, 95%, and 2 × 100%), cells were embedded in LX112. Ultrathin sections were stained with 1% uranyl acetate and lead citrate and then were examined for mitochondrial morphology on a H7000 electron microscope operating at 80 kV (Hitachi) [10].

### 2.6. Mitochondrial Membrane Potential Assay

Mitochondrial membrane potential (ΔΨm) was detected using a fluorescent probe, tetramethylrhodamine methyl ester (TMRM; Molecular Probes Inc., Leiden, Netherlands), which reversibly accumulates according to the membrane potential. Briefly, isolated hepatocytes and cardiomyocytes were incubated in HEPES buffer (134 mmol/L NaCl, 6 mmol/L KCl, 2 mmol/L CaCl_2_, 1 mmol/L MgCl_2_, 10 mmol/L HEPES, and 10 mmol/L glucose; pH 7.40) containing TMRM (100 nmol/L) for 30 min followed by a 15-minute wash. Flow cytometric analysis (BD FACSCalibur) was performed to analyze mean fluorescence intensity (MFI) [11].

### 2.7. Mitochondrial Respiratory Control Ratio

A commercially available kit (Genmed Scientifics Inc., Wilmington, DE, USA) was used to investigate mitochondrial respiratory control ratio (RCR) according to the manufacturer's instructions. The respiratory control ratio (RCR) was determined by calculating the ratio of State 3 to State 4. The RCR is a general measure of mitochondrial function and higher values denote better function. State 4 respiration is a model of steady-state, basal activity, while State 3 respiration is measured following the addition of ADP and mimics mitochondrial activity during exercise with high oxygen consumption and ATP turnover. The RCR was measured at 25°C. Briefly, mitochondrial fractions were incubated with Genmed reagent A. State 4 substrate solution (reagent B) and State 3 substrate solution (reagent C) were added in the proper sequence. Mitochondrial State 3 and State 4 respiration rates were determined and mitochondrial RCR was calculated as follows: RCR = State 3 respiration rate/State 4 respiration rate [12].

### 2.8. Measurements of the Intramitochondrial Free Ca^2+^ Concentration

The high-affinity cell-permeant Ca^2+^ indicator Fluo-4AM (Molecular Probes) was excited using short (about 1 s) flashes of light at 488 ± 10 nM and its emission was recorded using Flow Cytometry (BD FACS). Fluo-4AM was dissolved in DMSO and stored frozen (no more than a week) in the dark. Prior to the experiment, Pluronic F-127 was added to facilitate solution of Fluo-4AM in the aqueous (physiological) solution. In the working solution the final concentration of Fluo-4AM was 1 *μ*M, whereas the contents of DMSO and Pluronic F-127 were no more than 0.001%. The isolated preparation was stained with 1 *μ*M Fluo-4AM for 10 min at room temperature. Afterwards, the specimen was perfused with a physiological solution for 40 min, and measurements of the fluorescence were performed. Flow cytometric analysis (BD FACSCalibur) was performed to analyze mean fluorescence intensity (MFI).

### 2.9. Mitochondrial Reactive Oxygen Species Assay

H2DCFDA (2′,7′-dichlorodihydrofluorescein diacetate, Molecular Probes) was used as a cell-permeant indicator for ROS. The dye was excited using a 1 s flash of light at 480/15 nm every minute and its emission was recorded using Flow Cytometry (BD FACS). H2DCFDA was dissolved in DMSO (dimethyl sulfoxide). Pluronic F-127 (Molecular Probes) was used to enhance solution of the dye. The final concentration of DMSO and Pluronic F-127 in the working solution did not exceed 0.001%. The preparation was incubated with H2DCFDA (2 *μ*M) at room temperature for 15 min and after that it was perfused with physiological saline for 45 min before recording fluorescence intensity [13]. Flow cytometric analysis (BD FACSCalibur) was performed to analyze mean fluorescence intensity (DCF).

### 2.10. Mitochondrial Lipid Peroxidation Assay

The SOD activity was determined using SOD Assay Kit (Sigma-Aldrich, St. Louis, MO, USA). According to the provided protocol, the assay was based on utilization of Dojindo's highly water-soluble tetrazolium salt, WST-12-(4-iodophenyl)-3-(4-nitrophenyl)-5-(2,4-disulfophenyl)-2H-tetrazolium, monosodium salt, which produced a water-soluble formazan dye upon reduction with a superoxide anion. The rate of the reduction with O_2_ is linearly related to xanthine oxidase (XO) activity and is inhibited by SOD. The SOD activity as an inhibition activity was quantified by measuring the decrease in the color development at 450 nm. The SOD activity in the plasma was expressed as U/mg protein [14]. MDA is a sensitive marker of lipid peroxidation. A mitochondrial MDA assay was performed using a commercially available kit (Calbiochem, San Diego, CA, USA) according to the manufacturer's instructions. Briefly, mitochondrial fractions were resuspended in 5 mmol/L butylated hydroxytoluene. For each reaction, 200 *μ*L of mitochondria sample or standard was added to 650 *μ*L chromogenic reagents and 150 *μ*L of 12 N HCl at 45°C for 60 min. Samples were then centrifuged at 10,000 ×g for 5 min at 4°C and the supernatant was collected. The absorbance at 525 nm was recorded. The level of mitochondrial MDA was calculated using a standard curve.

### 2.11. Quantitative Reverse Transcription-Polymerase Chain Reaction

To quantitatively estimate PGC-1*α* and MnSOD mRNA expression in the rat's hepatocytes and cardiomyocytes, real-time polymerase chain reaction (RT-PCR) was performed using the following primer pair: PGC-1*α* forward primer 5′-CACCAAACCCACAGAGAACAG-3′ and PGC-1*α* reverse primer 5′-GCAGTTCCAGAGAGTTCCACA-3′; MnSOD forward primer 5′-CCAAGGGAGATGTTACAACTCAG-3′ and MnSOD reverse primer 5′-GGGCTCAGGTTTGTCCAGAA-3′. We used glyceraldehyde-3-phosphate dehydrogenase (GAPDH) as the internal control: GAPDH forward primer 5′-TGGTATCGTGGAAGGACTCATGAC-3′ and GAPDH reverse primer 5′-ATGCCAGTGAGCTTCCCGTTCAGC-3′. RNA isolation and conversion to complementary DNA (cDNA) were performed using a RevertAid First Strand cDNA Synthesis Kit (#K1622, Fermentas Inc., Hanover, MD, USA) following the manufacturer's instructions. cDNA suspension (1 *μ*g) was amplified with SYBR Green Quantitative PCR Master Mix (Applied Biosystems, Foster City, CA, USA). Assays were performed in triplicate with the ABI7500 instrument. The quantitative PGC-1*α* and MnSOD values for each cell line were estimated by dividing them by the GAPDH expression levels.

### 2.12. Statistical Analysis

Results are expressed as mean ± SEM. Two-way ANOVA was performed using SPSS17.0. Tests of equality of variance were carried out and post hoc tests were also performed. *P* value < 0.05 was considered statistically significant; ^*∗*^ *P* < 0.05; ^*∗∗*^ *P* < 0.01; ^*∗∗∗*^ *P* < 0.001.

## 3. Results

### 3.1. Salidroside Significantly Reduced the Mitochondrial Lesions

**Figure 1:**
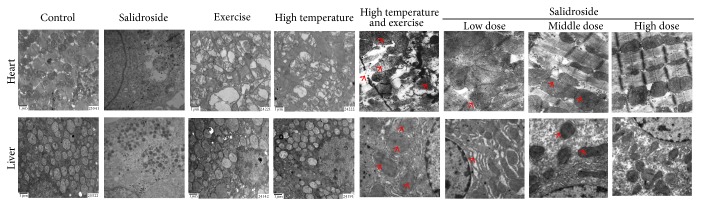
Salidroside suppressed EHS-induced morphologic alterations of mitochondria in cardiomyocytes and hepatocytes. Mitochondrial morphology in the EHS group and EHS + salidroside group (20, 50, and 100 *μ*M) was observed by an electron microscope (15,000x). The red arrowheads indicated the mitochondrial swelling, myelin breakdown, and disappearance of cristae matrix granules and, in mitochondrial matrix or concentric cristae, indicated the electron-lucent areas formed.

The animals were sacrificed and the tissues were taken when they stopped running [15]. There was water during heating.  Table 1 showed that the rats exercising in the heat suffered exertional heat stroke at exhaustion. The body temperature of the rats at that time was 46.2 ± 3.7°C in the EHS group rats. High temperature and exercise caused morphology changes of mitochondrial inner structure. As displayed in Figure 1, cardiomyocytes from EHS group rats were swelling with mitochondrial myelin breakdown and disappearance of cristae matrix granules. Also, electron-lucent areas were formed in mitochondrial matrix or concentric cristae (15,000x). Compared to the EHS group, mitochondrial lesions were attenuated in rats treated with salidroside at different concentrations (20, 50, and 100 *μ*M). Similar results were also obtained from hepatocytes.

### 3.2. Salidroside Restored EHS-Induced Decrease in ΔΨm

**Figure 2:**
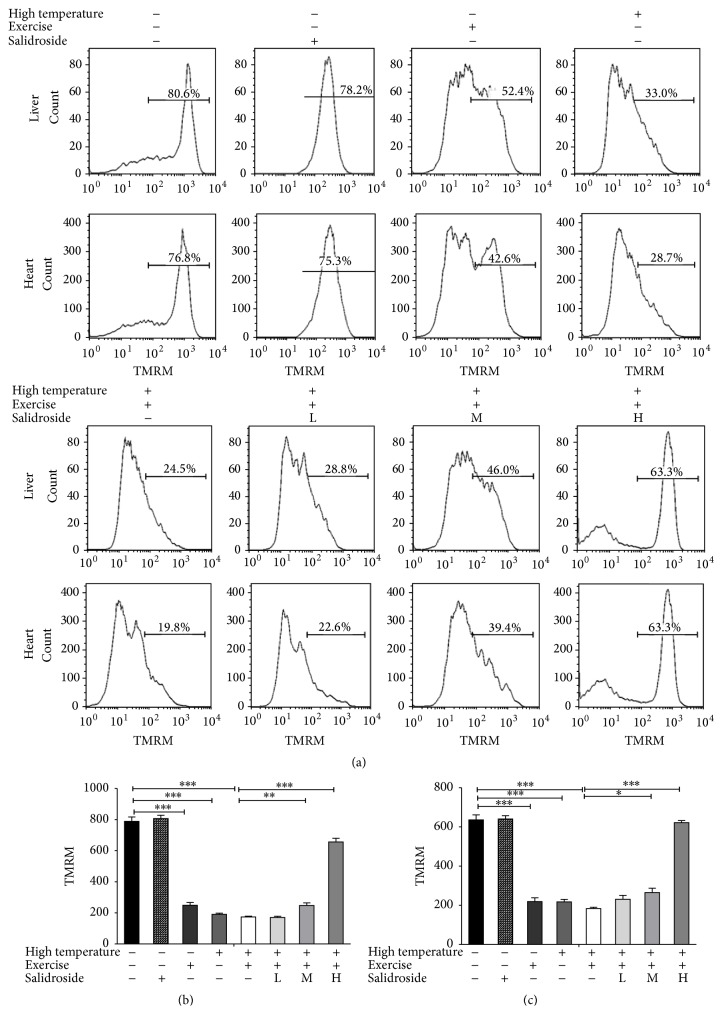
Salidroside restored EHS-induced decrease in ΔΨm. (a) ΔΨm (reflected by tetramethylrhodamine methyl ester) was assayed in hepatocytes and cardiomyocytes in the control group, high temperature group, exercise group, EHS group, and EHS + salidroside group (20, 50, and 100 *μ*M). (b) ΔΨm in hepatocytes in the control group, high temperature group, exercise group, EHS group, and EHS + salidroside group (20, 50, and 100 *μ*M). (c) ΔΨm in cardiomyocytes in the control group, high temperature group, exercise group, EHS group, and EHS + salidroside group (20, 50, and 100 *μ*M). Results were expressed as the mean ± SEM. *n* = 10. ^*∗*^ *P* < 0.05, ^*∗∗*^ *P* < 0.01, and ^*∗∗∗*^ *P* < 0.001.

Mitochondria are key regulators of cell death following alterations in ΔΨm in response to various triggers. ΔΨm in hepatocytes and cardiomyocytes was determined after treatment with high temperature, exercise, and/or various concentrations of salidroside (20, 50, and 100 *μ*M). A 79.4 ± 5.5% reduction in average fluorescent intensity incorporated into hepatocytes mitochondria was detected in EHS group (EHS group = 173.5 ± 7.8 versus control group = 788.5 ± 10.1, *P* < 0.001, Figure 2(b)) when compared to the control group. Consistent results were also obtained from cardiomyocytes with a 73.4 ± 6.7% reduction (EHS group = 183.0 ± 9.8 versus control group = 635.5 ± 6.6, *P* < 0.001, Figure 2(c)). ΔΨm was gradually increased after treatment with different concentrations of salidroside. After treatment with 100 *μ*M salidroside, ΔΨm in hepatocytes increased 71.6 ± 5.2% compared to the EHS group (salidroside group = 621.5 ± 6.3 versus EHS group = 173.5 ± 7.8, *P* < 0.001, Figure 2(b)). Similar results were also obtained from cardiomyocytes with a 69.9 ± 4.8% increase (Figure 2(c)).

### 3.3. Salidroside Improved RCR Decreased in EHS Rats

**Figure 3:**
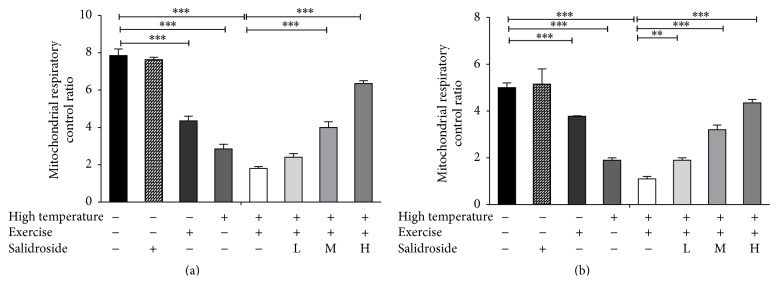
Salidroside increased the RCR. (a) RCR detected in hepatocytes in the control group, high temperature group, exercise group, EHS group, and EHS + salidroside group (20, 50, and 100 *μ*M). (b) RCR detected in cardiomyocytes in the control group, high temperature group, exercise group, EHS group, and EHS + salidroside group (20, 50, and 100 *μ*M). Results were expressed as the mean ± SEM. *n* = 10. ^*∗∗*^ *P* < 0.01, and ^*∗∗∗*^ *P* < 0.001.

The respiratory control ratio (RCR), a measure of the “tightness of coupling” between electron transport and oxidative phosphorylation, was determined from the ratio of State 3 to State 4 rates of respiration. In hepatocytes, the RCR was significantly decreased in exercise group, high temperature group, or EHS group compared with the control group (exercise group = 4.4 ± 0.3, high temperature group = 2.9 ± 0.4, and EHS group = 1.8 ± 0.1 versus control group = 7.8 ± 0.5, all *P* < 0.001, Figure 3(a)). However, the RCR was gradually improved in the salidroside groups with the increase of concentration when compared to EHS group (middle dose group = 4.0 ± 0.4 and high dose group = 6.4 ± 0.2 versus EHS group = 1.8 ± 0.1, all *P* < 0.001, Figure 3(a)). Consistent results were also obtained from cardiomyocytes (Figure 3(b)).

### 3.4. Salidroside Restored EHS-Triggered Decrease in Ca^2+^ Concentration

**Figure 4:**
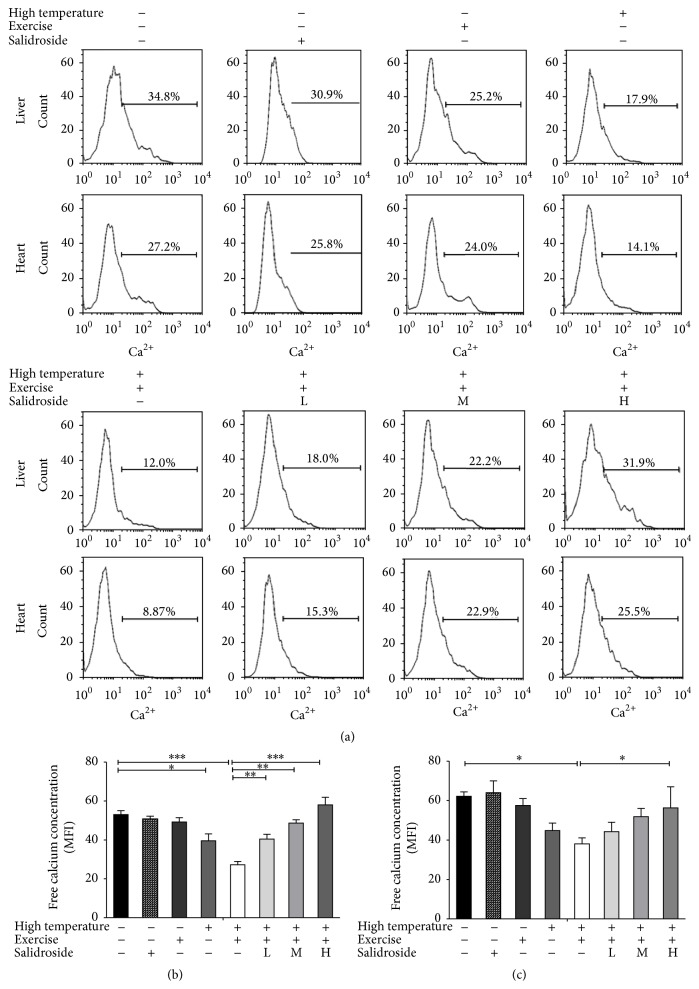
Salidroside restored EHS-triggered decrease in Ca^2+^ concentration. (a) Ca^2+^ concentration was detected by inverted fluorescence microscope in hepatocytes and cardiomyocytes in the control group, high temperature group, exercise group, EHS group, and EHS + salidroside group (20, 50, and 100 *μ*M). (b) Free Ca^2+^ concentration in hepatocytes in the control group, high temperature group, exercise group, EHS group, and EHS + salidroside group (20, 50, and 100 *μ*M). (c) Free Ca^2+^ concentration in cardiomyocytes in the control group, high temperature group, exercise group, EHS group, and EHS + salidroside group (20, 50, and 100 *μ*M). Results were expressed as the mean ± SEM. *n* = 10. ^*∗*^ *P* < 0.05, ^*∗∗*^ *P* < 0.01, and ^*∗∗∗*^ *P* < 0.001.

Ca^2+^ concentrations were measured after treatment with high temperature, exercise, and/or various concentrations of salidroside (20, 50, and 100 *μ*M). Compared with the control group, a 53.4 ± 3.5% reduction in average fluorescent intensity incorporated into hepatocytes mitochondria was detected in EHS group (EHS group = 27.3 ± 2.1 versus control group = 53.0 ± 2.8, *P* < 0.001, Figure 4(b)). Similarly, the average fluorescent intensity incorporated into cardiomyocytes mitochondria was reduced 36.1 ± 1.3% in EHS group (EHS group = 38.1 ± 4.3 versus control group = 62.2 ± 3.1, *P* < 0.05, Figure 4(c)). Ca^2+^ concentration was gradually increased after treatment with different concentrations of salidroside. After treatment with 100 *μ*M salidroside, Ca^2+^ concentration in hepatocytes increased 71.6 ± 5.2% compared to the EHS group (salidroside group = 57.9 ± 3.3 versus EHS group = 27.3 ± 2.1, *P* < 0.001, Figure 4(b)). Similar results were also obtained from cardiomyocytes with a 23.2 ± 1.6% reduction (Figure 4(c)).

### 3.5. Salidroside Reduced ROS Levels Caused by EHS Condition

**Figure 5:**
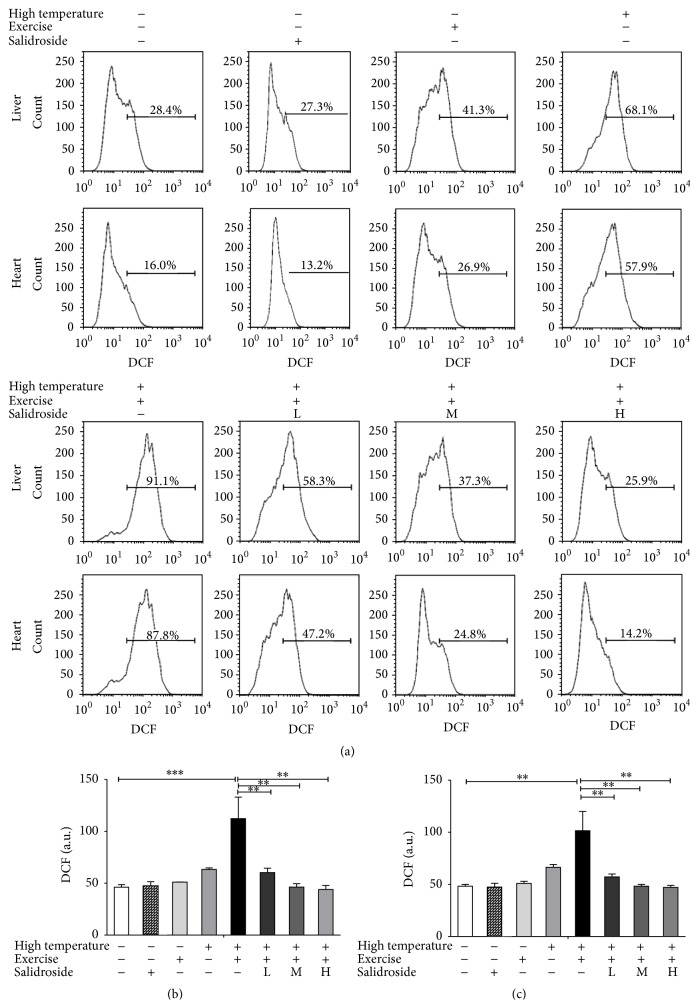
Salidroside inhibited excessive generation of ROS triggered by EHS. (a) ROS detected using specific fluorescent probes in hepatocytes and cardiomyocytes in the control group, high temperature group, exercise group, EHS group, and EHS + salidroside group (20, 50, and 100 *μ*M). (b) ROS level in hepatocytes in the control group, high temperature group, exercise group, EHS group, and EHS + salidroside group (20, 50, and 100 *μ*M). (c) ROS level in cardiomyocytes in the control group, high temperature group, exercise group, EHS group, and EHS + salidroside group (20, 50, and 100 *μ*M). Results were expressed as the mean ± SEM. *n* = 10. ^*∗∗*^ *P* < 0.01, and ^*∗∗∗*^ *P* < 0.001.

To investigate the effects of salidroside on EHS, ROS levels in hepatocytes and cardiomyocytes were evaluated using specific fluorescent probes for ROS. ROS production in hepatocytes was increased significantly in EHS group compared to that of the control group (EHS group = 112.3 ± 1.7 versus control group = 46.2 ± 0.9, *P* < 0.001, Figure 5(b)). However, the ROS levels were significantly reduced in salidroside groups (salidroside group = 43.9 ± 1.4 versus EHS group = 112.3 ± 1.7, *P* < 0.01, Figure 5(b)). Similar results were also obtained from cardiomyocytes (Figure 5(c)).

### 3.6. Salidroside Inhibited EHS-Induced MDA Activity Increase and Enhanced EHS-Triggered SOD Activity Decrease

**Figure 6:**
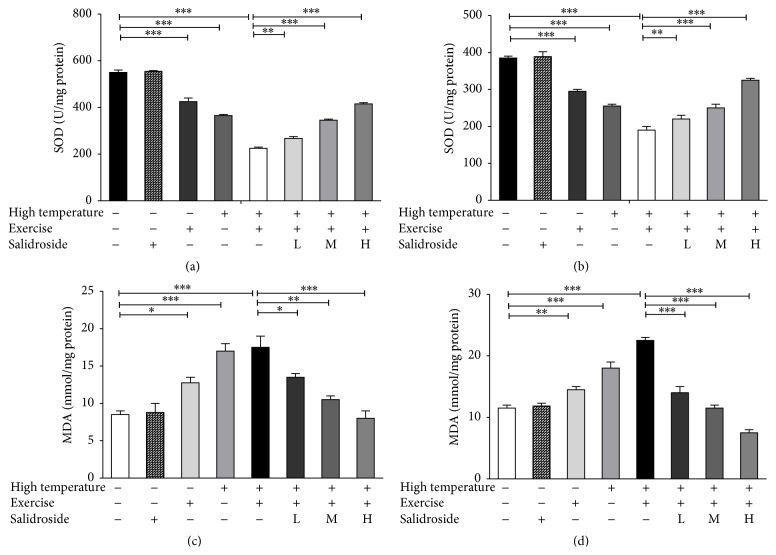
Salidroside inhibited EHS-induced MDA activity increase and enhanced EHS-triggered SOD activity decrease. (a) SOD activities in hepatocytes in the control group, high temperature group, exercise group, EHS group, and EHS + salidroside group (20, 50, and 100 *μ*M). (b) SOD activities in cardiomyocytes in distinct treatment groups. (c) MDA levels in hepatocytes in distinct treatment groups. (d) MDA levels in hepatocytes in distinct treatment groups. Results were expressed as the mean ± SEM. *n* = 10. ^*∗*^ *P* < 0.05, ^*∗∗*^ *P* < 0.01, and ^*∗∗∗*^ *P* < 0.001.

The activity of SOD decreased significantly in EHS group compared to that of the control group (*P* < 0.001). A significant decrease in SOD activity was also observed in the high temperature and exercise groups (*P* < 0.05 and *P* < 0.001, resp.). SOD activity was gradually increased after treatment with different concentrations of salidroside (Figures 6(a) and 6(b)). However, MDA activity was increased significantly in salidroside groups when compared to the EHS group. Increased endogenous MDA and decreased SOD indicated that the balance changed on the behalf of prooxidation in the liver homogenates of EHS rats.

### 3.7. Upregulation of PGC-1*α* and MnSOD in EHS Rats by Salidroside

**Figure 7:**
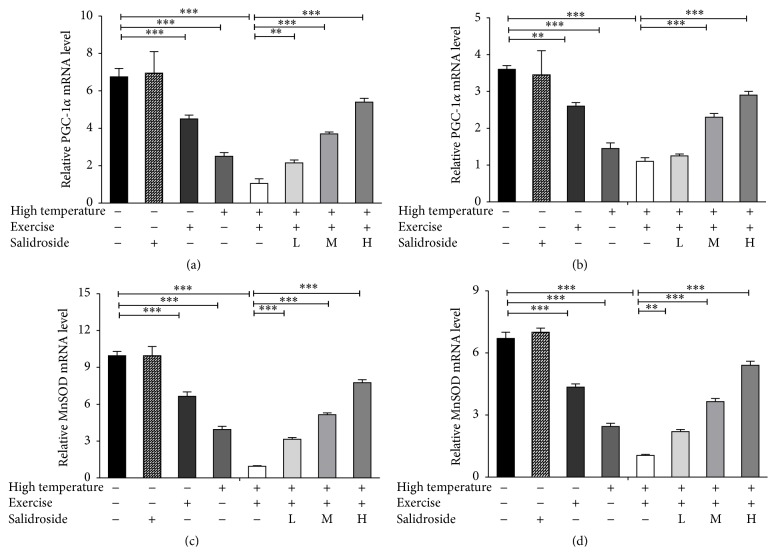
Salidroside reversed downregulation of PGC-1*α* and MnSOD stimulated by EHS. (a) Relative PGC-1*α* mRNA levels in hepatocytes in the control group, high temperature group, exercise group, EHS group, and EHS + salidroside group (20, 50, and 100 *μ*M). (b) Relative PGC-1*α* mRNA levels in cardiomyocytes in distinct treatment groups. (c) Relative MnSOD mRNA levels in hepatocytes in distinct treatment groups. (d) Relative MnSOD mRNA levels in hepatocytes in distinct treatment groups. Results were expressed as the mean ± SEM. *n* = 10. ^*∗∗*^ *P* < 0.01, and ^*∗∗∗*^ *P* < 0.001.

PGC-1*α* and MnSOD levels were assayed by RT-PCR method. The results showed the PGC-1*α* mRNA and MnSOD mRNA levels in hepatocytes and cardiomyocytes were downregulated in EHS group when compared to control group (Figure 7). However, the level of PGC-1*α* mRNA and MnSOD activity was increased significantly in salidroside groups when compared to the EHS group. This was consistent with recent data in that mitochondrial damage and PGC-1*α* downregulation are key events in MAO-A-dependent cardiomyocyte necrosis [16].

## 4. Discussion

EHS is a kind of severe illness characterized by central nervous system (CNS) abnormalities and potentially tissue damage resulting from elevated body temperatures induced by strenuous physical exercise and increased environmental heat stress. The high mortality rate of EHS is usually associated with multiorgan failure, especially coagulopathy and hepatic injury [17]. Both high temperature and hypoxia could impair mitochondrial function. The heart and liver are very susceptible to acute hypoxia or ischemia, because they are organs with high oxygen consumption [18]. Mitochondrial dysfunction included mitochondria swelling, ΔΨm decrease, ROS and MDA increase, and antioxidant enzyme SOD reduction. Appropriate regulation of mitochondrial biogenesis and function is a critical component of adaptation to external conditions and prevention of pathogenesis [19]. Salidroside has been demonstrated to have multiple biological effects [19–22]. In this study, we demonstrate that preservation of mitochondria function is the major mechanism for salidroside against EHS.

Loss of ΔΨm is a marker of mitochondrial dysfunction that ultimately leads to apoptosis. Mitochondria are key regulators of cell death following alterations in ΔΨm in response to various triggers. Our results demonstrated that ΔΨm reduced significantly in EHS rats, which is consistent with previous report which demonstrated that depletion of ΔΨm in EHS rats was early marker of the apoptotic process [18]. Moreover, we found that salidroside restored the depolarization of ΔΨm induced by EHS.

Our results showed that EHS caused significantly reduced Ca^2+^ concentration in hepatocytes and cardiomyocytes but induced a rapid and potent increase in ROS. Deregulation of intraneuronal Ca^2+^ is known to generate high levels of reactive oxygen species of mitochondrial origin (mt-ROS), a crucial step in the excitotoxic cascade [21]. The consequential role of ROS in the mechanism of cell cytotoxicity, especially the induction of apoptosis, has received increased attention in the field of EHS [22]. Salidroside was able to increase free Ca^2+^ concentration and reduce ROS production, thus relieving EHS damage.

An imbalance between production of ROS, oxidative damage to lipids (lipid peroxidation), and its elimination by the antioxidant defense system (such as SOD) in the body has been implicated for causes of aging and mitochondria related diseases [23]. Oxidative stress due to exhaustive training induced ROS increase in red gastrocnemius muscles, which led to a decrease in the cell antiapoptotic ability. Salidroside dramatically inhibited excessive generation of ROS by enhancing SOD activity, therefore protecting cardiomyocytes and hepatocytes against EHS-generated ROS attack, in accordance with the effect of salidroside to preserve, unaltered the mitochondrial ultrastructure as revealed by electron microscopy images. MDA, a product of lipid peroxidation, is elevated after EHS induction.

Moreover, we found that salidroside significantly enhanced SOD activity, while MDA activity was significantly decreased. These results suggested that the cardioprotective and hepatoprotective effects of salidroside might be related to the inhibition of ROS overgeneration and predisposing preservation of intact cells and integrity of mitochondria. PGC-1*α* is a transcriptional master coregulator of mitochondrial biogenesis, metabolism, and antioxidant defenses [24]. PGC-1*α*, which has been demonstrated to produce more uncoupled mitochondria, appears to have a stronger influence over antioxidant proteins, such as MnSOD and glutathione synthetic enzymes [5]. MnSOD is encoded by genomic DNA and its dismutase function is fully activated in the mitochondria to detoxify free radical O_2_^•−^ generated by mitochondrial respiration [25]. In our study, decreases of PGC-1*α* and MnSOD mRNA were found in hepatocytes and cardiomyocytes subjected to EHS. In contrast, salidroside improved PGC-1*α* and MnSOD mRNA and ameliorated such EHS-induced mitochondrial dysfunction.

In conclusion, we showed that salidroside exerts a protective effect against EHS-induced mitochondrial dysfunction and demonstrated that this occurs via inhibiting mitochondria swelling, preventing generation of ROS and MDA, and restoring ΔΨm, RCR, free Ca^2+^ concentration, SOD, PGC-1*α*, and MnSOD mRNA levels. More work in the future will be required to fully understand the beneficial role of salidroside against EHS and to further unfold the mystery of the mechanism involved.

## Figures and Tables

**Table 1:** Physical parameters of the rats.

Group	Time to exhaustion (min)	How far they ran (m)	The body temperature
Control	45.3 ± 1.1	203.4 ± 3.2	38.2 ± 1.3°C
Salidroside	42.1 ± 2.3	198.2 ± 2.1	38.5 ± 0.9°C
Exercise	35.3 ± 3.4	167.3 ± 1.1	42.7 ± 0.6°C
High temperature	36.2 ± 1.2	158.9 ± 2.9	43.4 ± 2.3°C
EHS	28.3 ± 2.3	129.2 ± 1.2	46.2 ± 3.7°C
Low dose	34.8 ± 2.1	147.8 ± 3.8	44.2 ± 1.7°C
Middle dose	38.9 ± 2.8	167.2 ± 2.7	40.5 ± 1.3°C
High dose	41.2 ± 3.2	188.3 ± 1.8	39.5 ± 0.7°C
